# Modeling biological gradient formation: combining partial differential equations and Petri nets

**DOI:** 10.1007/s11047-015-9531-4

**Published:** 2015-10-31

**Authors:** Laura M. F. Bertens, Jetty Kleijn, Sander C. Hille, Monika Heiner, Maciej Koutny, Fons J. Verbeek

**Affiliations:** 1 LIACS, Leiden University, Leiden, The Netherlands; 2 Mathematical Institute, Leiden University, Leiden, The Netherlands; 3 Department of Computer Science, Brandenburg Technical University Cottbus-Senftenberg, Cottbus, Germany; 4 School of Computing Science, Newcastle University, Newcastle Upon Tyne, UK

**Keywords:** Gradient formation, Petri net, Process validation, Quantitative modeling, Partial differential equation

## Abstract

Both Petri nets and differential equations are important modeling tools for biological processes. In this paper we demonstrate how these two modeling techniques can be combined to describe biological gradient formation. Parameters derived from partial differential equation describing the process of gradient formation are incorporated in an abstract Petri net model. The quantitative aspects of the resulting model are validated through a case study of gradient formation in the fruit fly.

In this paper we present a Petri net model of the biological process of gradient formation, incorporating parameters derived from a partial differential equation model of this process. In biology, a gradient is a graded change in concentration of specific signaling molecules, called morphogens, through a group of cells (Entchev and González-Gaitán 2002; Fischer et al. 2006; Gurdon et al. 1999; Gurdon and Bourillot 2001; Tomlin and Axelrod 2007). The morphogens get produced by a cell or group of cells, called the source, and emanate from there spreading throughout the tissue. At the same time molecules get degraded in the tissue. This simultaneous production and degradation establishes a slope in concentration levels, known as the morphogen gradient. Cells in the tissue sense the morphogen concentration in their direct surroundings and respond by adopting a specific behavior. In this way morphogens have a direct effect on cell development and differentiation and are therefore of the utmost importance (Wolpert 2002). For this reason, a model which furthers our understanding and analysis of the process, both from an operational as well as a denotational perspective, is of great use to the field of biology.

By combining a Petri net with parameters determined by a system of partial differential equations, we have constructed a generic Petri net model for the formation of molecular gradients.

An abstract proof of concept for the application of the Petri net framework to this biological phenomenon, has been presented in Bertens et al. (2012). This early model represents the process of gradient formation as a global decrease in concentration levels of molecules throughout the cells in the tissue. In the model, the spreading of molecules is governed by a fixed ratio of molecular concentration between neighboring cells. This ratio represents the combined effect of molecules being transported between cells and degrading in the cells.

In the current paper we present an elaboration of this model that makes it possible to include parameters derived from differential equation (DE) modeling. Starting from the proof of concept of Bertens et al. (2012), we move from an abstract approach towards a more detailed and applied approach. The events of molecule production, diffusion and degradation are modeled explicitly and are governed by individual parameters. For gradient formation, partial differential equation models exist which provide accurate quantitative data about this process (Gregor et al. 2005; Kicheva et al. 2007; Yu et al. 2009). By linking the parameters of the Petri net model to the parameters in the discretized form of such a DE model, the net can be used to produce quantitative data about discrete space and time points in the process, similar to the DE model, while at the same time retaining the advantages of the Petri net framework. In order to validate the Petri net model, we present a case study; from literature we have selected a study in which experimental observations of gradient formation have been modeled using partial differential equations. We use the parameters from this DE model and show how the simulation data obtained from executing the resulting Petri net correspond to the data obtained from the equations.

Both Petri nets and DE models have clear benefits for the study of biological processes (Ellner and Guckenheimer 2006; Gilbert and Heiner 2006; Gilbert et al. 2007; Heiner et al. 2008; Koch et al. 2011; Krepska et al. 2008; Matsuno et al. 2003; Steggles et al. 2006); by combining Petri nets and DEs we strive to bring characteristics of both together in one model. Many biological processes, in particular biochemical processes such as metabolic and signal transduction pathways, have been modeled using differential equations (Ellner and Guckenheimer 2006; Gregor et al. 2005; Kicheva et al. 2007; Yu et al. 2009). These mathematical models describe changes in process variables and enable precise quantitative studies, parameter sensitivity and bifurcation analysis. They assume the evolution of processes in continuous time and even continuous space. This allows the deduction of properties of the system mathematically, e.g. the existence and stability of steady states, by analyzing the system of DEs. Analysis is complemented by numerical simulations, to investigate the transient behavior, when the system is moving towards its long-term behavior. The simulation techniques involve discretization of the DEs, in time and space. The resulting computational scheme describes the change of state variables in discrete time steps.

On the other hand, the modeling framework of Petri nets (Petri 1962; Reisig 2010; Reisig and Rozenberg 1998), as an algorithmic process model aims to describe the mechanisms underlying (local) changes in a system (Ellner and Guckenheimer 2006; Priami 2009). Petri nets are moreover of particular use to biological studies, because of their origin in the modeling of chemical reactions and molecular interactions, and the explicit rendering of concurrent behavior, i.e. the independent and potentially simultaneous occurrence of events, which is a common feature of biological systems (Fischer et al. 2011; Koch et al. 2011). Futhermore, Petri nets combine graphical and mathematical elements, making them intuitive to communicate, execute and understand visually, while also allowing formal analysis. Implementation of a Petri net yields an operational process model. Using analysis tools such as state space exploration and analysis of, e.g., deadlocks and boundedness properties, the behavior of such a process can then be studied. In this way Petri nets provide a view point complementary to DE models. As for DE models, an interest in modeling biological processes with Petri nets has emerged, especially in the field of systems biology, and new ways to apply this modeling technique to the life sciences are constantly being developed (Banks 2009; Chaouiya 2007; Gilbert and Heiner 2006; Gilbert et al. 2007; Heiner et al. 2008; Koch et al. 2011; Krepska et al. 2008; Matsuno et al. 2003; Steggles et al. 2006). In Li and Yakota (2009), parameters for a Petri net representing bone remodeling are determined from a mathematical model for the biological process in terms of *ordinary* differential equations, whereas here we directly consider a partial differential equation. Furthermore, in Gilbert et al. biochemical processes evolving in time and space are considered with a spatial modeling approach which employs colored Petri nets for space discretization. For continuous models it corresponds to discretising partial differential equations. All analysis build on standard analysis/simulation techniques; e.g., the continuous Petri nets are simulated with standard ordinary differential equation solvers. In contrast, in this paper we present an alternative approach to solving partial differential equations using (discrete) Petri nets with an execution semantics based on the probably simplest time concept possible for this purpose.

It should be noted here that the Petri net model presented in this paper is not intended to just provide an alternative solution to a DE model of gradient formation. Rather it will be shown how parameters derived from the discretization of a PDE model for gradient formation provide quantitative information for an abstract Petri net model of this process. The resulting Petri net visualises the physical interaction on the level of particles (morphogens) and, where a DE model is based on global averages, the Petri net provides a view on local interactions between cells which offers new possibilities for a deeper understanding of gradient formation.

The paper is organized as follows. First, we give notions and notations related to Petri nets and we describe the modeling decisions. Subsequently, the discretization of the DE model is set out along with the connection of DE parameters to parameters in the Petri net modeling solution. Then we present the resulting Petri net model. A case study of gradient formation of the protein Dpp in the fruit fly is used for the validation. Finally, we present conclusions and remarks on future work.

The work presented in this paper was carried out as part of the PhD research of the first author (Bertens 2012).

## Preliminaries

### PT-nets with activator arcs

For a general introduction to Petri nets we refer to Reisig and Rozenberg (1998). In this paper, we use Place/Transition-nets with activator arcs (Kleijn and Koutny 2007), PTA-nets for short, and a maximally concurrent execution rule (Burkhard 1983).

Petri nets are defined by an underlying structure with *places* and *transitions* as basic elements, connected by directed, *weighted arcs*. In the Petri net model considered in this paper, there are moreover *activator arcs* connecting places to transitions. In modeling, places are usually the passive elements, representing local states, and transitions the active elements. Here, global states, referred to as *markings*, are defined as mappings assigning to each place a natural number (of *tokens* corresponding to available resources).

A *PTA-net* is a tuple $N=(P,T,W, Act ,m_0)$ such that:

- *P* and *T* are finite disjoint sets of *places* and *transitions*, respectively.
- $W:(T\times P)\cup (P\times T)\rightarrow \mathbb {N}$ is the *weight function* of *N*.
- $Act \subseteq P\times T$ is the set of *activator* arcs of *N*.
- $m_0:P\rightarrow \mathbb {N}$ is the *initial marking* of *N*.

**Fig. 1:**
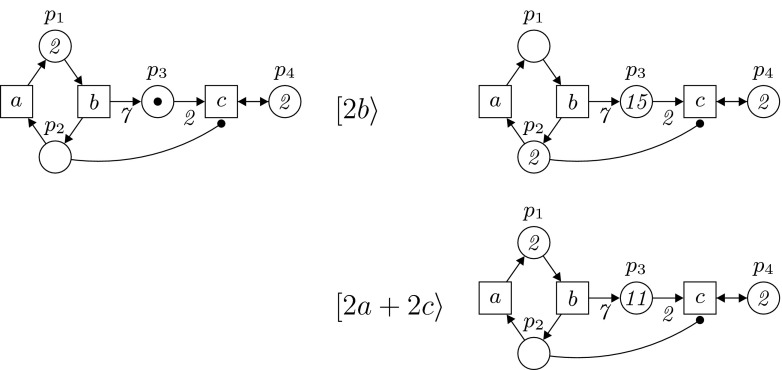
A PTA-net *N* and its evolution $N[2b\rangle N'[2a+2c\rangle N''$ generating the max-enabled step sequence $(2b)(2a+2c)$. Note that we use integers rather than tokens to represent markings greater than 1

In diagrams, such as that shown in Fig. 1, places are drawn as circles, transitions as boxes, and arcs are arrows. If $W(x,y) \ge 1$, then (*x*, *y*) is an *arc* leading from *x* to *y*; it is annotated with its weight if this is greater than one. Activator arcs have black-dot arrowheads. A marking $m$ is represented by drawing in each place *p* exactly $m(p)$ tokens as small black dots, or just inserting there the integer $m(p)$. We assume that each transition *t* has at least one input place (there is at least one place *p* such that $W(p,t)\ge 1$).

When a single transition *t* occurs (‘fires’) at a marking, it takes tokens from its input places and adds tokens to its output places (with the number of tokens consumed/produced given by the weights of the relevant arcs). Moreover, if there is an activator arc $(p,t)\in Act$, then transition *t* can only be executed at the given marking if *p* contains at least one token, without the implication of tokens in *p* being consumed or produced when *t* occurs. Thus, the difference with a *self-loop*, *i.e.* an arc from *p* to *t* and vice versa, is that the activator arc only tests for the presence of tokens in *p* without requiring exclusive access rights to these tokens during firing.

We define the executions of *N* in more general terms of simultaneously occurring transitions. A *step* is a multiset of transitions $U:T\rightarrow \mathbb {N}$. Thus *U*(*t*) specifies how many times transition *t* occurs in *U*. (Note that if we exclude the empty multiset, single transitions can be considered as minimal steps.) A non-empty multiset *U* can be written in the form of a formal sum $U(t_1) t_1 + \cdots + U(t_n) t_n$ if $T = \{t_1, \ldots , t_n\}$ and if $U(t_i)$ is 0, the term $0 t_i$ is skipped. Step *U* is *enabled* (to occur) at a marking $m$ if $m$ assigns enough tokens to each place for all occurrences of transitions in *U* and, moreover, all places tested through an activator arc by a transition in *U*, contain at least one token.

Formally, step *U* is enabled at marking $m$ of *N* if, for all $p\in P$:

- $$m(p)\ge \sum _{t\in T}U(t)\cdot W(p,t)$$
- $m(p)\ge 1$ whenever there is a transition *t* such that $U(t)\ge 1$ and $(p,t)\in Act$.

If *U* is enabled at $m$, it can be *executed* leading to the marking $m'$ obtained from $m$ through the accumulated effect of all transition occurrences in *U*:

- $$m'(p)=m(p)+\sum \nolimits _{t\in T}U(t)\cdot (W(t,p)-W(p,t))$$ for all $$p\in P$$.

Finally, a step *U* is said to be *max-enabled* at $m$ if it is enabled at $m$ and there is no step $U'$ which is also enabled at $m$ and strictly contains *U* (meaning that $U'\ne U$ and $U(t)\le U'(t)$ for all transitions *t*). We denote this by $m[U\rangle m'$. A (max-enabled) *step sequence* is then a sequence $\sigma =U_1 \ldots U_n$ of non-empty steps $U_i$ such that $m_0 \,[U_1\rangle \, m_1\, \ldots \, m_{n-1}\,[U_n\rangle \,m_n$, for some markings $m_1,\ldots ,m_n$ of *N*. Then $m_n$ is said to be a *reachable* marking of *N* (under the maximally concurrent step semantics). Figure 1 depicts a max-enabled step sequence.

This particular net model was chosen in Bertens et al. (2012) to describe the formation of a gradient for the following reasons. First of all, it follows from the above definitions that the chosen Petri net semantics (the rules for the execution of steps) allows *auto-concurrency*, the phenomenon that a transition may be executed concurrently with itself. This approach makes it possible to use transitions for a faithful modeling of natural events like the independent (non-sequential) occurrence in vast numbers of a biochemical reaction in a living cell. Note that the degree of auto-concurrency of a transition can easily be controlled by a dedicated place with a fixed, say *k*, number of tokens connected by a self-loop with that transition implying that never more than *k* copies of that transition can fire simultaneously.

Activator arcs are a means of *testing* for the presence of at least one token in a place (see, e.g., Kleijn and Koutny 2007), and so they are similar to other kinds of net features designed for the same reason. We mentioned already self-loops by which the presence of a token in a place can be tested by a single transition which ‘takes and returns’ the token, but not simultaneously by an arbitrary number of transition occurrences in a step. Two other mechanisms which do allow such multiple testing are *context* arcs (Montanari and Rossi 1995) and *read (or test)* arcs (Vogler 2002). Activator arcs are however more permissive since they only check for the presence of a token *before* the step is executed (this is often referred to as *a priori* testing). We feel that *a priori* testing is more appropriate for biological applications as the ‘lookahead’ implied by the other two kinds of test arcs is hard to imagine in reality.

Finally, the *maximal* concurrency in the steps that are executed, reflects the idea that execution of transitions is never delayed. This may also be viewed as a version of time-dependent Petri nets where all transitions have a firing duration of 1. Moreover, applying maximal concurrency in this paper, was inspired by Petri nets with *localities* (Kleijn et al. 2006) and their associated *locally maximal* semantics. Here one may think of, e.g., the locally synchronous occurrence (in pulses) of reactions in individual compartments of a cell. Such an approach, based on localities of activities, seems also appropriate when various aspects of a developmental process are to be modeled.

### Modeling decisions

We choose to use cells as the elementary units in our model, represented by places. Tokens represent morphogen levels, conducted from cells to neighboring cells by the transitions. Tokens can represent exact molecule numbers, as is the case in our validation, or a limited range of semi-qualitative concentration levels. This is a relevant characteristic, since biological gradients often work in a rather discrete, semi-qualitative manner; a number of cell responses (such as activation of a particular gene) exists for a given gradient and threshold values in morphogen concentration demarcate the boundaries between these responses, resulting in a stepwise change in cellular behavior throughout the tissue. Due to this, both semi-qualitative and quantitative ways of modeling can represent biological situations realistically; our Petri net model is applicable to both.

Our model focuses on local signaling between neighboring cells. In the biological situation, the number of morphogens to be transported from one cell to the next depends solely on the difference in morphogen level between these two neighboring cells; cells have no ’knowledge’ of morphogen transport in other parts of the tissue. In order to accurately reflect this situation we base the computation of transported tokens solely on the difference in token numbers between the neighboring cells. This makes the model easily scalable, i.e. the number of cells in the tissue is irrelevant to the computation and can be adjusted without altering the workings of the model.

With these biological decisions in mind we have opted to use concurrent steps rather than individually occurring transitions. Morphogen transport between cells is not directly influenced by events taking place in non-adjacent cells, which means these processes should be able to take place concurrently and non-adjacent cells can be simultaneously involved in the transport of morphogens. Moreover, since in the biological situation morphogens move to the next cell as soon as this is possible, we have chosen to use maximal enabled steps for our net semantics.

Instead of merely calculating the final distribution of the tokens, we want our net to model the gradual process of morphogen movement through the tissue, i.e. to represent also intermediate steps. This results in an operational description of the behavior of the system, which will allow the user to simulate experiments in which the biological process is altered while running; e.g., grafting experiments, in which parts of the tissue get removed or replaced, can be simulated by taking cells out of the net at a certain moment during execution.

## Derivation of Petri net model parameters from the discretized DE model

In this section the temporally and spatially continuous situation, modeled by a DE model, is translated to a discrete situation, which is subsequently linked to the Petri net solution. We consider the following reaction-diffusion equation

1 $$\begin{aligned} \frac{\partial C}{\partial t} = D\frac{\partial ^2 C}{\partial r^2} - k C \end{aligned}$$

on the one-dimensional interval (0, *L*). This is used in the case study in Kicheva et al. (2007) as the effective equation to describe their data. It reflects the measurement of fluorescence of GFP-labeled morphogens when these form a gradient in a rectangular sample of cell tissue. The morphogens are homogeneously emanating from a source, which is a strip of cells at the left border ($r=0$) of this rectangle. The morphogens move from the source to the right, i.e. towards $r=L$. The fluorescence measurements are made in multiple vertical layers in the tissue and summed, reducing the situation to two dimensions. The morphogen concentration can be assumed constant in the direction transversal to *r*, further reducing the situation to one dimension. Thus, *C*(*r*, *t*) represents the areal density of observed morphogen at location *r* at time *t*. *D* is the effective diffusion coefficient ($\upmu$m$^2$/s), combining passive diffusion and possible other transport processes such as endocytosis and active diffusion, and *k* is the degradation rate (s$^{-1}$). Equation (1) is complemented with an initial condition $C(r,0)=f(r)$ and zero-flux boundary conditions at *L* and constant influx areal density $J_0$ through the left side of the sample at $r=0$, *i.e.* $D\frac{\partial }{\partial r}C(0)=-J_0$.

The standard procedure of spatial discretization at equidistant points $0=r_0<r_1<\dots <r_n=L$, with $\ell =r_{i+1}-r_i$ and $C_i(t):=C(r_i,t)$, followed by temporal discretization at time points $t_j$, in which *j* represents the number of steps and the steps are equally separated at time intervals $\Delta t$ (corresponding to a fixed number of $n'$ steps) yields

2 $$\begin{aligned} \frac{\Delta C_i(t_j)}{\Delta t} \approx \frac{D}{\ell ^2} \bigl ( C_{i-1}(t_j)- 2C_i(t_j) + C_{i+1}(t_j) \bigr ) \ -kC_i(t_j) \end{aligned}$$

for $i=1,\dots , n-1$, where $\Delta C_i(t_j):= C_i(t_{j+1})-C_i(t_j)$. We take $\ell$ equal to the cell length and *h* to the cell height. Multiplying both sides of (2) with the cell area $A=\ell h$ in the plane of observation yields a similar, slightly rewritten expression for the change in the number $m_i=m_i(t_j)$ of molecules in cell *i* at time $t_j$ (omitting time dependence):

3 $$\begin{aligned} \Delta m_i \approx \frac{D\Delta t}{\ell ^2}\bigl ( m_{i-1} - m_i\bigr ) - \frac{D\Delta t}{\ell ^2}\bigl ( m_{i} - m_{i+1}\bigr ) - k\Delta t\, m_i \end{aligned}$$

for $i=1,\dots , n-1$, with $\Delta m_i=m_i(t_{j+1})-m_i(t_j)$. Approximation (3) is appropriate when $\Delta t$ and $\ell$ are such that $\frac{D\Delta t}{\ell ^2}<1$ and $k\Delta t<1$ are sufficiently small. Equation (3) is complemented by similar equations at $i=0$ and $i=n$ that incorporate the boundary conditions:

4 $$\begin{aligned} \Delta m_0&= J_0 h\Delta t \ -\ \frac{D\Delta t}{\ell ^2}\bigl ( m_{0} - m_1\bigr ) \ -\ k\Delta t\, m_0 \end{aligned}$$

5 $$\begin{aligned} \Delta m_n&= \frac{D\Delta t}{\ell ^2}\bigl ( m_{n-1} - m_n\bigr ) \ -\ k\Delta t\, m_n\;. \end{aligned}$$

If we now consider a Petri net with the maximally concurrent step semantics and a sequence $x_1,\ldots , x_n$ of places representing the biological cells, the equations above correspond to the three main events in the process of gradient formation in the following manner: the first term on the right hand side of (4) represents morphogens being produced in the source and transported to the first cell, $x_1$; the transport between neighboring cells $x_i$ and $x_{i+1}$ is given by the first two terms on the right hand side of (3), while the degradation in every $x_i$ is given by the third term on the right hand side of (3). In other words, the marking of the places $x_i$ (for all places except $x_1$) after $jn'$ steps can be approximated well by the solution of the diffusion equation (1) at times $t_j=j\Delta t$:

6 $$\begin{aligned} m_i(jn')&\approx h\int _{(i-1)\ell }^{i\ell }C(r,t_j)\,dr \end{aligned}$$

7 $$\begin{aligned}&\approx \ell h\cdot \frac{1}{2} \bigl [ C((i-1)\ell ,t_j)\;+\; C(i\ell ,t_j)\bigr ] \end{aligned}$$

where we have used the trapezium rule to approximate the integral. In this way we relate the molecule number $m_i$ to the marking of place $x_i$, i.e. $m(x_i)$. This brings us to the Petri net solution and its exact workings.

## Modeling solution

**Fig. 2:**
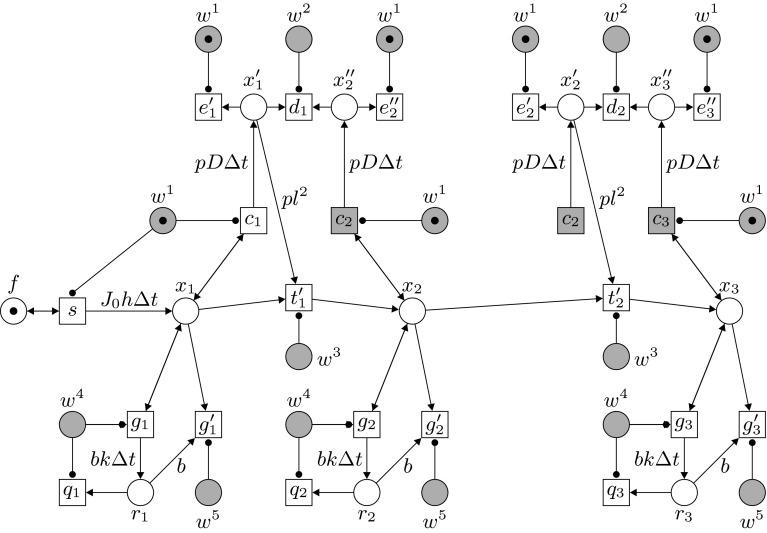
The main construction of the net, shown for the first three neighboring cells. Note that the *grey places* and transitions are fusion elements

The previous section illustrated the discretization of the DE model and how to link the resulting parameters for production, transport and degradation to a Petri net model. In this section we present our Petri net model and give a detailed account of its dynamics. We propose a formal, general Petri net model for gradient formation. Given is a segment of *n* adjacent biological cells with the *i*-th cell as the immediate neighbor of the $(i + 1)$-st cell. This is represented in the Petri net by places $x_1,\ldots , x_n$. Morphogens are represented by tokens and can be transported only between immediate neighbors. Transitions $t'_1,\ldots ,t'_{n-1}$ represent the transport of tokens, in the direction $x_1$ to $x_n$. We will focus on one-directional gradient formation, strictly from $x_1$ to $x_n$. Figure 2 shows the basic structure of the net; here the first neighboring cells on the left side of the modeled biological tissue are shown as $x_1$, $x_2$ and $x_3$. (Places and transitions with the same name should be identified; such fusion elements are shown in grey.) Above we discussed the derivation of parameters from differential equations for three basic elements in the process. Here we explain the way in which these parameters are incorporated into the Petri net model.

1. Morphogen **production** and transport from the source to the adjacent cell $x_1$ are modeled by the transition *s* and comply to the first term of (4), which is directly translated to the weight $J_0 h \Delta t$ of the arc from *s* to $x_1$.
2. The morphogen **transport** from $x_i$ to $x_{i+1}$ follows the first term on the right hand side of (3), which corresponds to the effective diffusion from left to right. In order to incorporate this term into the Petri net, additional, auxiliary places $x'_1,\ldots ,x'_{n-1}$ and $x''_2,\ldots ,x''_n$ are used. These places are initially empty. Through the simultaneous and maximal concurrent firing of transitions $c_i$ ($1 \le i \le n$), all places $x'_i$ where $1 \le i \le n-1$, are filled with $p D\Delta t \cdot m(x_i)$ tokens and all places $x''_i$ where $2 \le i \le n$, with $p D \Delta t \cdot m(x_i)$ tokens. Here we have introduced a new constant *p* which is used later to control the accuracy of the computation and handle rounding errors. Next all places $x'_i$ and $x''_{i+1}$ ($1 \le i < n$) are depleted simultaneously by transitions $d_i$, emptying $x''_{i+1}$ and leaving $x'_i$ with a token difference of
  $$\begin{aligned} pD\Delta t \cdot m(x_i) - pD\Delta t \cdot m(x_{i+1})\;. \end{aligned}$$
  Here we use that $m(x_i) \ge m(x_{i+1})$ for every reachable marking *m* if initially place $x_i$ contains no less tokens than $x_{i+1}$. The number of tokens to be transported from $x_i$ to $x_{i+1}$ is
  $$\begin{aligned} \beta _i = \frac{D\Delta t \cdot m(x_i) - D\Delta t \cdot m(x_{i+1})}{l^2} \end{aligned}$$
  in other words, for every $l^2$ tokens in place $x_i$ one token is to be moved by transition $t'_i$ from $x_i$ to $x_{i+1}$, respectively. In the Petri net this is implemented using the constant *p*: for every $pl^2$ tokens in place $x'_i$ a token is moved from $x_i$ to $x_{i+1}.$
  The steps described here correspond directly to Eq. (3) without the element of degradation (to be discussed below), as can be seen from the following:
  8 $$\begin{aligned} m'(x_i)= & {} m(x_i) - \beta _i + \beta _{i-1} \nonumber \\= & {} m(x_i) - \frac{pD\Delta t \cdot m(x_i) - pD\Delta t \cdot m(x_{i+1})}{pl^2} \nonumber \\&+ \frac{pD\Delta t \cdot m(x_{i-1}) - pD\Delta t \cdot m(x_i)}{p l^2} \nonumber \\= & {} m(x_i) - \frac{D\Delta t}{l^2} (m(x_i) - m(x_{i+1})) \nonumber \\&+ \frac{D\Delta t}{l^2} (m(x_{i-1}) - m(x_i)) \end{aligned}$$
3. Simultaneously with morphogen transport, morphogen **degradation** also takes place in the cells, which corresponds to the third term on the right hand side in (3). For every $x_i$, this process is modeled by the transitions $g_i$ and $g'_i$ and the place $r_i$, which is again an auxiliary place used to determine the number of tokens to be removed from $x_i$. The place $r_i$ is filled through the maximal concurrent occurrence of $g_i$, with $b k \Delta t\cdot m(x_i)$ tokens; multiplication with *b* is used to prevent having to round off $k \Delta t$, since due to the small value of *k* for most biological gradients, this will often lead to 0. Subsequently, since $k \Delta t \le 1$, for every *b* tokens in $r_i$, a token from $x_i$ disappears. This results in a degradation $\frac{b k \Delta t}{b} = k \Delta t$, which corresponds with the third element on the right hand side in (3).

**Fig. 3:**
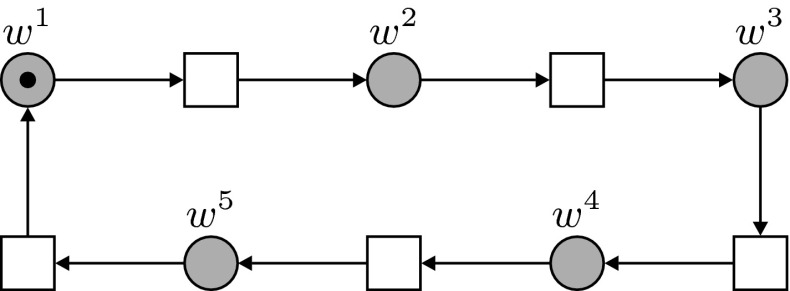
The auxiliary construction of the net, determining the order of execution in the main net. Note that the *grey places* are fusion places

These processes of production, transport and degradation take place in a cycle of 5 steps. An auxiliary net, shown in Fig. 3, is used to regulate these phases and the corresponding transitions. This net is similar to the auxiliary net employed in Bertens et al. (2012); in this earlier model, degradation was not modeled in explicit steps (diffusion and degradation were combined in one parameter) and the cycle was limited to three steps. The auxiliary net controls the transitions via five places $w^1-w^5$, and activator arcs. For the full picture of the system one should identify (fuse) all places with the same name in Figs. 2 and 3 (where these fusion places are shown in grey). In the auxiliary net a token moves cyclically from one place $w^j$ to the next and consequently the events in the main net are scheduled in the following order, with the number of a step corresponding to the number of the place *w* which contains the token at that point:

1. For $1 \le i \le n$, transition $c_i$ fills in $m(x_i)$ auto-concurrent occurrences, place $x'_i$ (if $i < n$) and place $x''_i$ (if $i > 1$) with $p D \Delta t \cdot m(x_i)$ tokens. In the same step, transitions $e'_i$ and $e''_{i+1}$ empty $x'_i$ and $x''_{i+1}$ of any residual tokens left from the previous cycle. In addition, if place *f* contains a token, transition *s* outputs $J_0 \cdot \Delta t \cdot h$ tokens to $x_1$.
2. Transition $d_i$ removes tokens from places $x'_i$ and $x''_{i+1}$ in $m(x''_{i+1})$ auto-concurrent occurrences, thereby emptying $x''_{i+1}$ and leaving the difference $\alpha$ in $x'_i$; in other words, in the resulting marking $m'$ we have $m'(x'_i) = \alpha = pD\Delta t \cdot m(x_i) - pD\Delta t \cdot m(x_{i+1})$.
3. Transition $t'_i$ fires and transports $\frac{\alpha }{p l^2}$ tokens from $x_i$ to $x_{i+1}$.
4. In the steps corresponding to $w^4$ and $w^5$, the degradation of morphogens in the individual cells is addressed. In step 4 transition $g_i$ inserts $b k \Delta t \cdot m(x_i)$ tokens into place $r_i$. Simultaneously, transition $q_i$ empties $r_i$ of any residual tokens left from the previous cycle.
5. Subsequently, transition $g'_i$ removes one token from $x_i$ for every *b* tokens present in $r_i$.

The auxiliary net regulates the five phases of the computational process which determines for all locations the number of morphogens moving from one cell to the next, the actual transport, and the amount of degradation. Places representing neighboring pairs of cells are either all involved in calculation steps or tokens are transferred between them or disappear. During the computation steps (1, 2, and 4), the token numbers in all places $x_i$, except place $x_1$, are not changed and their current number of tokens can be checked by other transitions. In other words the computational process is orthogonal to the basic operations of gradient formation. Another important feature of this approach is that it is purely local; interactions between neighboring cells are independent of the token numbers in other cells or the length of the chain of cells. Due to the maximal auto-concurrency semantics, the Petri net exhibits a fully concurrent behaviour. Interactions between the cells and the passing of tokens representing particles (morphogens) take place everywhere as soon as locally possible and such interactions are completely independent of interactions taking place elsewhere.

As we will demonstrate later, the Petri net model is a computational implementation of observations of gradient formation (as recorded in a PDE model). Only the places $x_i$ and their markings, modeling cells and morphogens, reflect a biological reality. The rest of the net performs a computational process to realize the diffusion process of morphogen transport between cells. Thus, *e.g.*, places $x_1'$, $x_2''$ do not correspond to biological substances, but are local counters and provide the input to the local calculations by the Petri net.

## A case study of Dpp gradient formation to validate the Petri net model

For a validation of the Petri net model we use data presented in Kicheva et al. (2007). In this study, gradient formation was examined for the protein Dpp (Decapentaplegic) in the wing of the fruit fly, *Drosophila melanogaster*. The protein was studied as it emanated from a source through the wing epithelium. The gradient could be treated as a series of physical localities. The 3D situation was captured in a stack of images. Firstly, a maximum projection of this stack reduced the tissue to a two-dimensional plane; this could further be reduced to a line of places, since the rectangular region of interest lay parallel to the rectangular source tissue and movement at the lateral sides was negligible.

Kicheva et al. studied the behavior of gradient formation and the role played in this by the process of endocytosis, *i.e.* the uptake of particles through membrane vesicles into the cell, which is known to contribute to the formation of many gradients, in addition to diffusion (Gilbert and Heiner 2006; Gurdon and Bourillot 2001; Lander et al. 2002; Scholpp and Brand 2004; Teleman et al. 2001). To this end the authors created a partial endocytotic block in animals which were mutant for the *shibire* allele and in which the source was rescued by a $\textit{shibire}^{+}$ transgene. Using an experimental set-up, monitoring fluorescent recovery after photobleaching (known as a FRAP assay), the values for *D* and *k* were determined under different experimental conditions of the gradient formation. Here we simulate the gradient formation for the Dpp *shibire* mutant at 32$^\circ$C (Dpp-rescue) and the Dpp control group at 32$^\circ$C (Dpp).

For these conditions the following values for *D* and *k* were found by Kicheva et al. (2007) and used here in the Petri net model presented (omitting the standard deviation): for Dpp $D = 0.10$ and $k = 2.52$ and for Dpp-rescue $D = 0.06$ and $k = 1.53$. Based on these values, values for *p* and *b* were set at $p = 10^2$ and $b = 10^5$, in order to minimize rounding errors. The simulation results from the Petri net model were compared to those predicted by the DE model, using the experimentally determined parameter values for *D*, *k*, *l*, $j_0$ and *h* as found by Kicheva et al. (2007). For this validation the number of cells to be modeled has been set at 30, which is a large enough number to accurately model *L*, given the current case study. The Petri net therefore describes the situation of a linear array of 30 cells, with a constant influx of morphogens at the left ($r=0$). At the far right side we assumed that morphogens cannot flow out of the last cell. In our DE model this is represented by zero flux boundary conditions at $r=L$ (see above; $L=30l$). Note that this differs from the DE model employed in Kicheva et al., where the array of cells is assumed to extend infinitely far. This has consequences for the exact solution at steady-state and the time-dependent solutions.

Gradient formation is considered to be finished once a steady state has been reached, i.e. a state in which morphogen concentrations stay the same in all cells, due to a balance between production, diffusion and degradation. For the diffusion equation model, the exact steady-state solution $C^*$ to the diffusion equation (1) with infinitely extending array of cells ($L=\infty$) is given by

9 $$\begin{aligned} C^*(r) = \frac{j_0}{\sqrt{kD}}\, e^{-\mu r},\qquad \mu :=\sqrt{\frac{k}{D}}. \end{aligned}$$

In our case, with a finite array of cells and Neumann conditions at $r=L$, (9) requires an additional correction factor: the exact steady state solution becomes

10 $$\begin{aligned} C^*(r) = \frac{j_0}{\sqrt{kD}}\, e^{-\mu r} \,\cdot \, \frac{1+e^{2\mu (x-L)}}{1-e^{-2\mu L}}\;. \end{aligned}$$

The time-dependent solutions in both cases will start to differ once morphogens have reached the end at $x=L$ in sufficient amounts. In the case of a finite array these morphogens will start influencing the flux at positions $x<L$, which will not happen in the infinitely extended case, because then they escape to infinity. For a proper comparison between the partial differential equation model and the Petri net these boundary effects have been taken into account in (10).

For comparison of the density description by means of *C* with the number of tokens in a cell as computed by the Petri net, we convert the first to the number $N_k$ of morphogen molecules in cell *k*, by means of

11 $$\begin{aligned} N_k(t) := h\int _{(k-1)l}^{kl} C(r,t)dr, \end{aligned}$$

where *l* denotes the cell length, *h* the cell height and $k=1,2,\dots ,30$. The integral in (11) is approximated by means of the basic trapezoidal rule, yielding

12 $$\begin{aligned} N_k(t) \approx hl\cdot \frac{1}{2} \bigl [ C((k-1)l,t) + C(kl,t)\bigr ]\;. \end{aligned}$$

Here $$l=2.6$$ $$\upmu$$m and $$h=2.6$$ $$\upmu$$m.

In the Petri net a steady state is reached once the marking of the entire net after two consecutive step cycles is the same. This is because the net is deterministic and the parameter values remain unchanged. For each of the experiments the Petri net solution with corresponding parameter values was implemented in the software tool Snoopy (Rohr et al. 2010), which in its latest version also supports constants. The markings of the places $x_1,\dots , x_n$ for every 5 steps (the step cycle) were obtained using our in-house analysis tool PetriCalc. Snoopy was used as an interface for the creation of the net, but due to the size of the net and the high numbers of tokens to be processed, analysis was done with PetriCalc. The steady state as reached by the Petri net for Dpp and Dpp-rescue was found to closely correspond to the steady state given by (10) and (12), with only minor deviations: at most 1.4 % for Dpp-rescue and 0.2 % for Dpp.

**Fig. 4:**
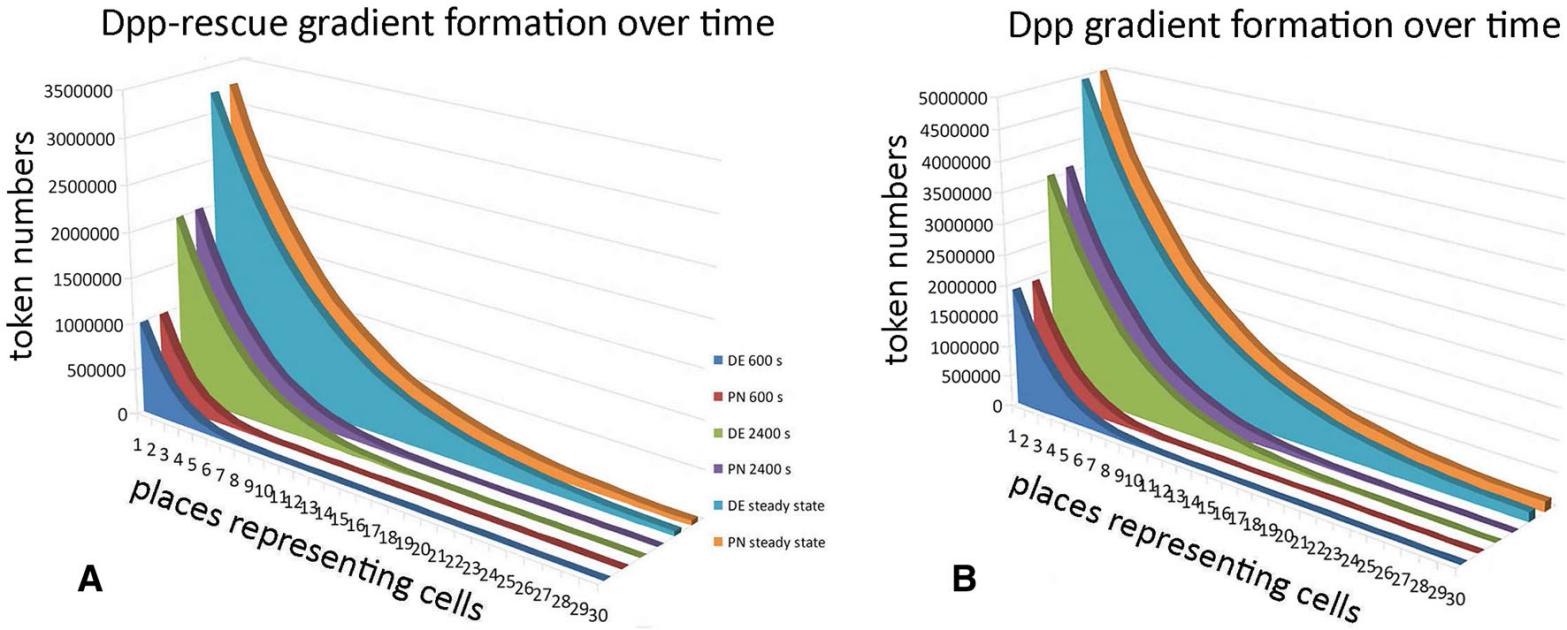
Visualization of different stages in the process of gradient formation

In addition to the steady state, we also compared the gradient formation at $t= 600$ s and $t=2400$ s. For the DE model, these time-dependent solutions were computed using the finite element package^1^ COMSOL Multiphysics (version 4.2.0.150). Again the Petri net and the DE model yielded corresponding results, with minor deviations: on average 0.01 % for Dpp with a maximum of 0.46 % and on average 0.02 % for Dpp-rescue with a maximum of 0.2 %. In Fig. 4, the Petri net marking corresponding to times $t= 600$ s, $t=2400$ s and *t* = 12,000 s are compared to the values predicted by the DE model; the situation at *t* = 12,000 s represents the steady state.

## Conclusion and discussion

We have presented a Petri net model for biological gradient formation based on a transfer of parameters of a DE model to a Petri net structure with the aim to describe the local changes within the process. The model is generic in the sense that it has parameters that can be instantiated on basis of concrete PDE systems describing gradient formation. The quantitative aspects of the model have been validated through a case study of Dpp and Dpp-rescue gradient formation in the fruit fly.

The combination of DE models and Petri nets as proposed in this paper leads to an alternative point of view on the process modeled. Implementing a DE description of a biological phenomenon in a PN model makes it possible to move from a macroscopic, global approximation of a biological process to a description closer to physical interactions and subprocesses. In general, this method requires an understanding of the process concerned in terms of causes and effects (modeled in the Petri net) that would fit observations captured in a DE model.

In this paper, the biological process is gradient formation described in the form of a one-dimensional reaction-diffusion equation which provides the parameters for an abstract Petri net modeling local relations within the process. Whereas the PDE model is based on global averages, the Petri net takes the spatial discreteness of the cellular tissue into account from the start. It visualises the physical interaction on the level of particles (morphogens) and provides a view on local interactions between cells. It does not assume knowledge of PDEs and how to solve them. Transient solutions are replaced by easier to manipulate sequences of marked places. Moreover, we have shown that the distribution of markings at discrete time points of the Petri net corresponds to the values obtained for the PDE model. Thus the Petri net model facilitates and supports the implementation, simulation, and visualisation of different scenarios and their effect on gradient formation and offers new possibilities for a deeper understanding of this process. One could, e.g., investigate what-if scenarios by running the net for a new situation. Differences in morphogen uptake and release characteristics, tissue inhomogeneities, either natural or experimentally induced, can be more easily modeled in the Petri net model than in the PDE model where the cellular spatial structure is lacking.

The current model is amenable to experimental set-ups which interfere with the unfolding of the process. It is possible to simulate for instance grafting experiments, in which part of the tissue is removed or replaced and the effects are studied. For gradient formation in particular, experiments have been performed with fluorescence recovery after photobleaching (FRAP; Carrero et al. 2003; Kicheva et al. 2007). In such experiments part of a tissue containing fluorescently labeled proteins is locally photobleached, after which recovery of the gradient of fluorescence is studied. Since the structure of the presented Petri net closely resembles the observed biological situation, it can be used to simulate such experiments, by removing places which correspond to particular biological cells or depleting these of tokens. Similarly, one can investigate what happens in case of bounded cell capacities by blocking or leaking tokens in the Petri net model.

**Fig. 5:**
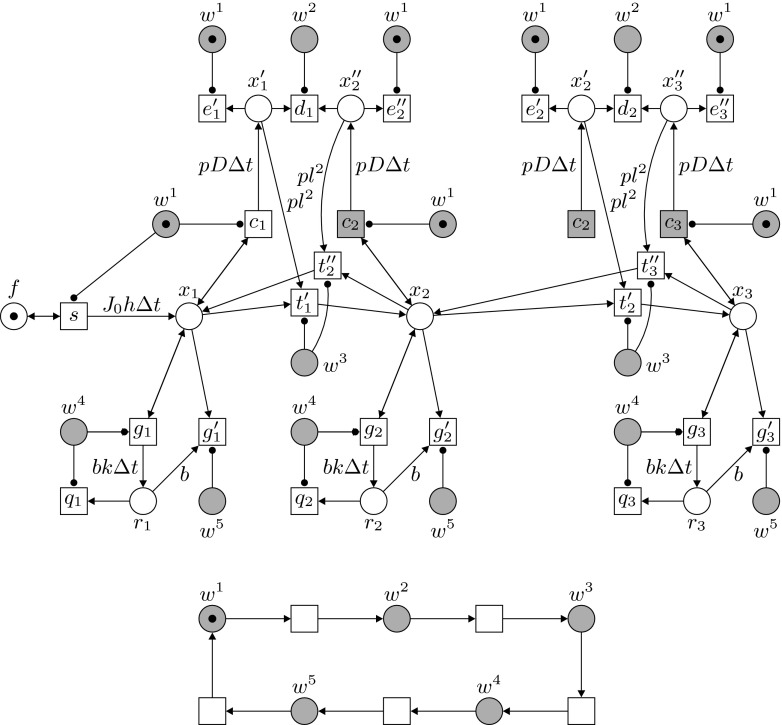
The main construction of the net and auxiliary net, in the case of two-directional gradient construction

While the current model represents the tissue as a one-dimensional structure, i.e. a line of cells, the approach is amenable to extension in two and three dimensions. Again this potential is due to the combined strength of the formalisms; while DEs enable the user to easily compute the steady state of a gradient system, this becomes increasingly difficult when multiple spatial dimensions are included. Each added dimension results in additional boundary conditions, making computations highly complex. In contrast to this, the spatial arrangement of places in a Petri net can be extended relatively easily, to include more dimensions. We are currently investigating the adaptation of the Petri net, to model 2-dimensional cell layers and 3-dimensional tissues. Furthermore, by adding one more chain of transitions $t''_2,\ldots ,t''_n$, the Petri net can be made to model transport of tokens between neighboring cells in two directions, as illustrated in Fig. 5. A Petri net with bi-directional transport could be calibrated and then validated for a DE system and corresponding case describing such process (when at hand).

The model is versatile and due to its modular nature, it can easily be adjusted to a variety of instances of gradient formation, with regard both to changes in parameter values and to the length of the tissue under study (and folded into a colored Petri net, see Gilbert et al.). As a combination of PDEs with a Petri net, the model offers a wide range of possibilities for analysis and in silico experiments. Similar to DE models, it allows quantitative analysis of gradient formation. Thus the possibilities of Petri nets and DE models complement each other, yielding a powerful framework for the study of gradient formation.

Since gradients play a pivotal role in developmental biology, the development of tools for the description and analysis of this process, e.g., in embryology, is of great value. Using an hierarchical approach like the one exemplified in Viana de Carvalho et al. (2015) the Petri net modeling gradient formation could be integrated into a hierarchical net model together with (Petri net) models of other processes underlying or regulating gradient formation. As future work, we also hope to explore other possibilities of building hierarchical nets, using for instance nets-within-nets (Valk 2004) and/or refinement, to model particular subcellular processes of gradient formation, such as passive and active diffusion through the extracellular space and degradation by means of endocytosis.

## Footnotes

1 Ideally, one would like to compare the changes in markings in the Petri net over discrete time steps with an exact time-dependent solution of the PDE model. Since a tractable analytic expression for the latter is not available, we used a readily available and well-accepted numerical simulation tool to compute the time-dependent solution which uses a finite element scheme to get a good approximation of the true solution of the PDE model.
